# ATAC-seq normalization method can significantly affect differential accessibility analysis and interpretation

**DOI:** 10.1186/s13072-020-00342-y

**Published:** 2020-04-22

**Authors:** Jake J. Reske, Mike R. Wilson, Ronald L. Chandler

**Affiliations:** 1 Department of Obstetrics, Gynecology and Reproductive Biology, College of Human Medicine, Michigan State University, Grand Rapids, MI 49503 USA; 2 Center for Epigenetics, Van Andel Research Institute, Grand Rapids, MI 49503 USA

**Keywords:** ATAC-seq, Chromatin accessibility, Bioinformatics, Normalization, Genomics, Differential accessibility

## Abstract

**Background:**

Chromatin dysregulation is associated with developmental disorders and cancer. Numerous methods for measuring genome-wide chromatin accessibility have been developed in the genomic era to interrogate the function of chromatin regulators. A recent technique which has gained widespread use due to speed and low input requirements with native chromatin is the Assay for Transposase-Accessible Chromatin, or ATAC-seq. Biologists have since used this method to compare chromatin accessibility between two cellular conditions. However, approaches for calculating differential accessibility can yield conflicting results, and little emphasis is placed on choice of normalization method during differential ATAC-seq analysis, especially when global chromatin alterations might be expected.

**Results:**

Using an in vivo ATAC-seq data set generated in our recent report, we observed differences in chromatin accessibility patterns depending on the data normalization method used to calculate differential accessibility. This observation was further verified on published ATAC-seq data from yeast. We propose a generalized workflow for differential accessibility analysis using ATAC-seq data. We further show this workflow identifies sites of differential chromatin accessibility that correlate with gene expression and is sensitive to differential analysis using negative controls.

**Conclusions:**

We argue that researchers should systematically compare multiple normalization methods before continuing with differential accessibility analysis. ATAC-seq users should be aware of the interpretations of potential bias within experimental data and the assumptions of the normalization method implemented.

## Introduction

Genome-wide quantitative sequencing methods for measuring genomic features have been recently developed to address various biological questions previously limited to locus-level interrogation. Current applications include gene expression [1], DNA methylation [2], protein–DNA interactions [3], histone post-translational modifications [4], 3D genome organization [5], nucleosome occupancy [6] and chromatin accessibility [7]. Researchers frequently apply these techniques to multiple cellular states in parallel to provide biological insight into the questions being investigated. These approaches remove experimental predispositions by permitting genome-wide analysis paired with robust statistical testing procedures to improve null hypothesis rejection. As a result, there is an obvious need to benchmark and improve statistical or analytical methods used for properly interpreting the sequencing data generated by the molecular biology.

The latest major technique for effectively measuring genome-wide chromatin accessibility is the Assay for Transposase-Accessible Chromatin, or ATAC-seq. This assay makes use of a Tn5 transposase reaction which preferentially inserts a 9-bp adapter fragment into accessible regions of the genome [8]. Adapter-ligated genomic regions, which are typically nucleosome-depleted and euchromatic, can then be enriched for sequencing. This technique provides a similar readout as DNase I hypersensitivity (DNase-seq) and formaldehyde-assisted isolation of regulatory elements (FAIRE-seq), which also measure accessible chromatin regions, and it is an orthogonal assay to Micrococcal nuclease digestion (MNase-seq), which measures nucleosome-occupied regions [9, 10]. However, ATAC-seq offers many benefits over comparable assays including a lower input material requirement, shorter assay time, in situ library preparation, and further protocol adaptation to fresh-frozen tissue [11]. These advantages have permitted precise in vivo regulatory genomic assays on small populations of sorted cells [12–17].

ATAC-seq has been used to both identify basal accessible chromatin regions in a given cellular context as well as determine regions differentially accessible (DA) between two cellular states [18–20]. The former of which is analyzed bioinformatically through a linear process which often involves calling signal peaks throughout the genome. In this respect, the analysis framework for ATAC-seq is similar to ChIP-seq and DNase-seq [21], though few comprehensive analyses and best practice reports exist. Previous work has evaluated performance of computational methods in DNase-seq footprinting analysis, and similar efforts should be made for ATAC-seq [22]. For DA analysis, ATAC signal at enriched regions is quantified and compared between multiple conditions. Determining DA regions with high confidence poses a greater challenge due to variability in transposition reaction efficiency, upon which may be further compounded by in vivo heterogeneity and lack of guiding literature. The result is that outputs from multiple tools for calculating DA regions are often conflicting. Furthermore, while there are indeed a small number of studies which have attempted to streamline ATAC-seq data processing [23–26], there is little emphasis on statistical considerations of differential analysis.

Using our recently reported in vivo sorted mouse ATAC-seq data set, we show that different tools and normalization methods for calculating significant DA regions yield distinct results following disruption of a chromatin remodeler [17]. We show that qualitative techniques like MA plots (as applied in microarray analysis) can be used to identify and address global accessibility patterns which may or may not be technical in nature. We assess the DA outputs from 8 different analytical approaches and observe vastly different numbers of significant genome-wide DA regions, promoter DA regions, and global accessibility trends depending on the approach used. By cross-comparing DA method outputs, we are able to define genes commonly identified as having a DA promoter across multiple approaches. Integrating RNA-seq data allowed us to determine biological relevance of each method by assessing overlap between promoter DA and differential gene expression. Next, we analyze an ATAC-seq data set by Schep et al. [18] and show that choice of DA method can alter biological interpretation. We also implemented negative control DA analysis in these data to highlight the sensitivity of our analysis in distinguishing signal from noise. We then propose a generalized ATAC-seq data processing workflow intended for DA analysis and supply a detailed step-by-step guide which includes example code and scripts for users. This framework includes key steps for robust comparison which reduce upstream biases, such as differing molecular complexities between libraries. We further test the ability of this workflow to identify biologically relevant peaks on a historic data set reported by original authors of this method, Buenrostro et al. [8], and show it is effective in these data. Finally, we propose a differential accessibility R workflow through *csaw* which permits testing of multiple normalization methods. In whole, researchers should be aware of differing biological interpretations resulting from different normalization methods and any biases, which may not be considered or eliminated during analysis. This is especially true wherein widespread chromatin structure alterations might be expected, such as when disrupting chromatin regulators. We further provide computational methodology that serves as a “one size fits all” guideline for ATAC-seq data analysis from reads to differential accessibility analysis.

## Results

### Comparison of 8 analytical approaches to calculate ATAC-seq differential accessibility

To determine if choice of ATAC-seq DA analysis method influences experimental results, we compared 8 different DA approaches (Table 1) using the published tools *MACS2*, *DiffBind*, *csaw*, *voom*, *limma*, *edgeR*, and *DESeq2* [27–33]. Analyses *I* and *II* follow the *DiffBind* protocol, originally intended for ChIP-seq data, which constructs a consensus read count matrix from *MACS2* replicate peak sets of *m* query regions by *n* samples. Briefly, *MACS2* constructs an ATAC fragment pileup from aligned paired-end data, then builds a local bias track through a series of parameters to estimate background noise, and finally compares ATAC signal to the local background at each genomic bp using a Poisson test. Significant nearby regions are then merged into a peak. *DiffBind* then calculates linear scaling factors from either the total number of reads in each library, which assumes that true global differences may be expected and technical bias is small, or the total number of reads in queried peak regions, which should eliminate global differences in favor of reducing any technical biases. The former method is applied in *I* and the latter in *II*. The count matrix with normalization factors is then subject to the *DESeq2* framework of dispersion estimation and negative binomial generalized linear model (GLM) fitting for hypothesis testing, according to the design matrix. Analyses *III* through *VI* follow approaches described in the *csaw* manual [28]. *III* and *IV* count reads in query regions defined by *MACS2* peak sets then filter low-abundance windows, while *V* and *VI* use the *csaw* sliding window approach to quantify ATAC signal in the 300-bp interval query windows across the genome. The de novo query windows in *V* and *VI* then pass low-abundance filtering and are tested for signal enrichment greater than threefold over the surrounding 2 kilobase local neighborhood. For normalization, *III* and *V* implement the trimmed mean of *M* values (TMM [34]) method to generate linear scaling factors from counts in large, 10-kb genomic bins. This method trims the top and bottom quantiles of bins based on fold-change and signal abundance in order to minimize the changes between samples at the majority of bins. TMM assumes that most regions are not truly DA, and it assesses for systematic signal differences present across the genome that are presumed to be technical. Therefore, the TMM method should control for technical error more than scaling to total read depth by eliminating any systematic biases in library ATAC distribution, while still permitting true asymmetric differences specifically in DA regions. *IV* and *VI* implement a non-linear loess-based (loess: locally estimated scatterplot smoothing) normalization method. This highly conservative method normalizes the signal distribution locally based on extent of ATAC signal abundance. As a result, the loess fit assumes a symmetric global distribution in which there are no true biological global differences in ATAC reaction efficiency or distribution, and any evidence of these biases are technical and should be removed. The count matrices with respective normalization factors or offsets from all four of these *csaw* analyses are then subject to the *edgeR* statistical framework of estimating dispersions by empirical Bayes and quasi-likelihood GLM fitting for hypothesis testing, according to the design matrix. Finally, analyses *VII* and *VIII* follow the same procedure as *III* and *IV* with respect to using *MACS2* peak query regions filtered by abundance in *csaw*, but instead they follow a *voom* transformation to log_2_ counts per million (log_2_CPM) in *VII* which are further quantile normalized in *VIII*. The log_2_CPM transformation simply scales by full library size and maintains those assumptions, while quantile normalization equalizes the signal distribution across all libraries [35]. Quantile normalization should function analogously to loess normalization by eliminating any global or trended biases, and it has been previously applied to ATAC-seq data [20]. The (normalized) log-count matrices from these two analyses are then mean–variance estimated to generate weights for *limma* linear modeling and hypothesis testing by empirical Bayes-moderated statistics.

**Table 1:** Description of 8 approaches used to calculate ATAC-seq differential accessibility

*#*	Genomic regions	Tool	Normalization	DA testing
*I*	*MACS2* [27]	*DiffBind* [29]	Full library size	DESeq2 [31]
*II*	*MACS2* [27]	*DiffBind* [29]	Reads in peaks	*DESeq2* [31]
*III*	*MACS2* [27]	*csaw* [28]	TMM [34]	*edgeR* [30]
*IV*	*MACS2* [27]	*csaw* [28]	Loess	*edgeR* [30]
*V*	*csaw* [28]	*csaw* [28]	TMM [34]	*edgeR* [30]
*VI*	*csaw* [28]	*csaw* [28]	Loess	*edgeR* [30]
*VII*	*MACS2* [27]	*csaw* [28] | *voom* [32]	Log_2_cpm	*limma* [33]
*VIII*	*MACS2* [27]	*csaw* [28] | *voom* [32]	Quantile [35]	*limma* [33]

### Choice of ATAC-seq analytical approach is a key step in determining differential chromatin accessibility

We recently reported an ATAC-seq data set in which chromatin accessibility was compared between sorted mutant and control mouse endometrial epithelial cells following disruption of a common tumor suppressor and oncogene [17]. In this in vivo study, a chromatin remodeler protein, ARID1A, was disrupted along with induced expression of a constitutively active oncoprotein, PIK3CA^H1047R^, resulting in myometrial [17] and peritoneal [36] invasion in *LtfCre*^*0*/+^; (*Gt*)*R26*^Pik3ca*H1047R^; *Arid1a*^*fl*/*^ mutant mice. Mutant and wild-type endometrial epithelial cells were positively selected by a surface marker, EPCAM, and purified by magnetic bead separation [17]. ATAC-seq was selected as a suitable method for analyzing chromatin accessibility changes in sorted cells due to feasibility of supplying low input material.

**Fig. 1:**
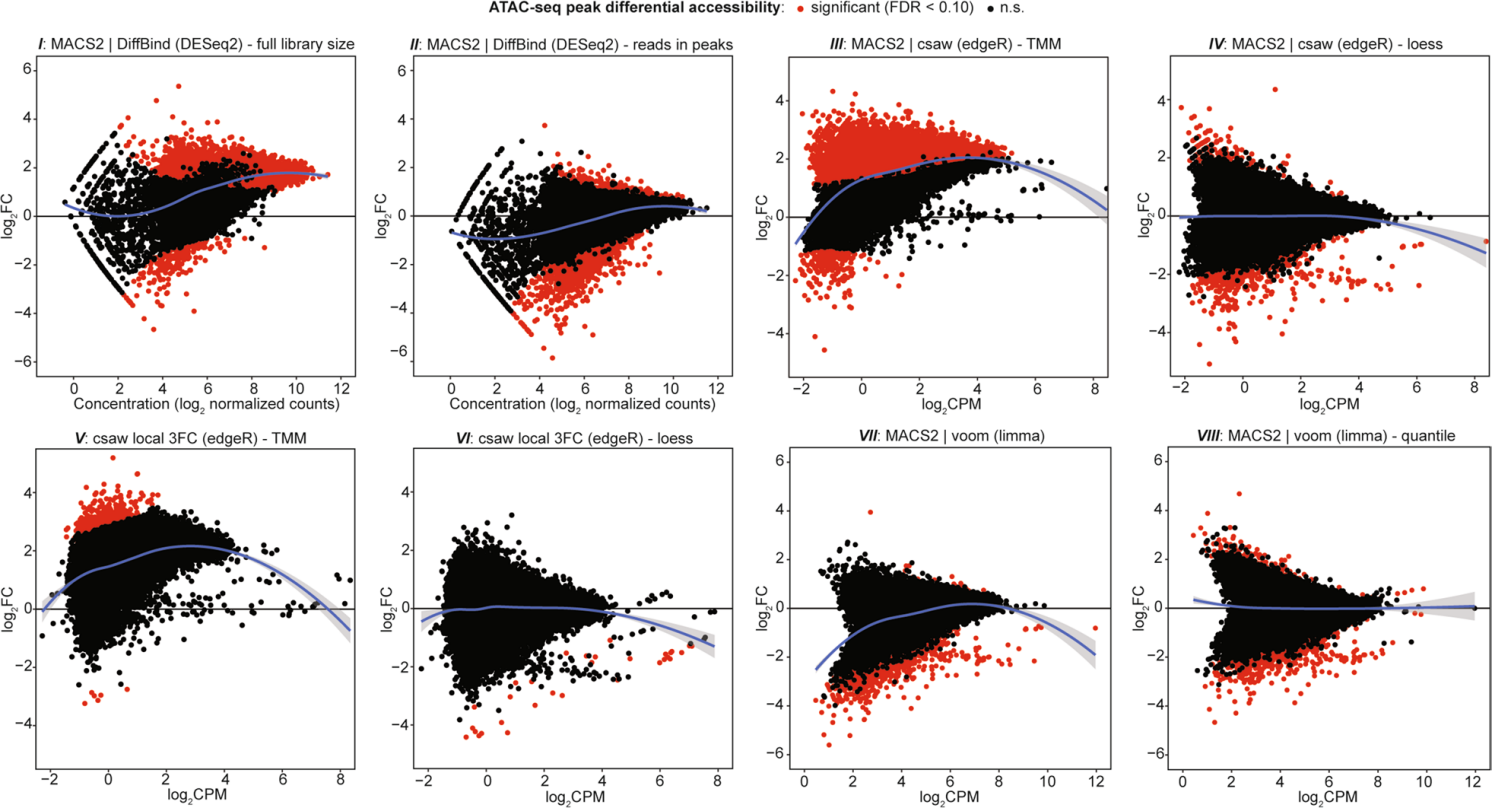
DA distributions from the same ATAC-seq data set analyzed by 8 different DA approaches. Example MA plots for ATAC-enriched regions of interest analyzed for differential accessibility by different approaches. *I* and *II* are from *DiffBind* using *MACS2* peak sets and with scaling factors derived from full libraries or reads in peaks only, respectively. *III* and *IV* are from *csaw* using *MACS2* peak sets as query regions with either a TMM or non-linear loess-based normalization method. Likewise, *V* and *VI* are from *csaw*, but instead using de novo query regions identified through local neighborhood enrichment. *VII* was calculated using MACS2 peak sets transformed to log_2_ counts per million (log_2_CPM) by *voom* which is further quantile normalized in *VIII*. MA plot *X*-axis represents average ATAC signal abundance at that region, while *Y*-axis is the log_2_ difference in ATAC signal between the two conditions. Black dots represent non-significant regions, and red dots represent significant (FDR < 0.10) DA regions. Blue lines are loess fits to each distribution with 95% confidence intervals shaded in gray

We next compared chromatin accessibility patterns between mutant and control endometrial epithelial populations using the 8 DA approaches. Different patterns of DA measurements were observed through MA plot visualization depending on the choice of DA approach used (Fig. 1). MA plots are a type of Bland–Altman plot applied to genomic data, where they were originally used in microarray analysis [37]. MA plots depict global patterns of measurements compared between two samples, where each tested genomic feature is quantified by the difference between the two groups as the *y*-axis (*M*) and signal intensity as the *x*-axis (*A*). For example, TMM normalization of the *MACS2* peaks read count matrix in *III* shows the majority of these genomic regions are increasing accessibility in mutant cells compared to control cells, whereas a small minority are decreasing. The TMM approach is similar to the default normalization method of *DiffBind* in *I* which scales counts based on library size, but TMM only considers regions expected to be unchanged for generating normalization factors. These observations are in contrast with the DA results of a more conservative loess-based count adjustment in *IV*. The loess normalization yielded more significant DA regions that were decreasing rather than increasing accessibility. MA plots for DA regions following all varying analyses show how these global patterns are affected through different normalization methods. The DA distribution is shifted upwards in the TMM normalized windows, which may or may not be technical in nature, and is corrected with the loess normalization.

**Fig. 2:**
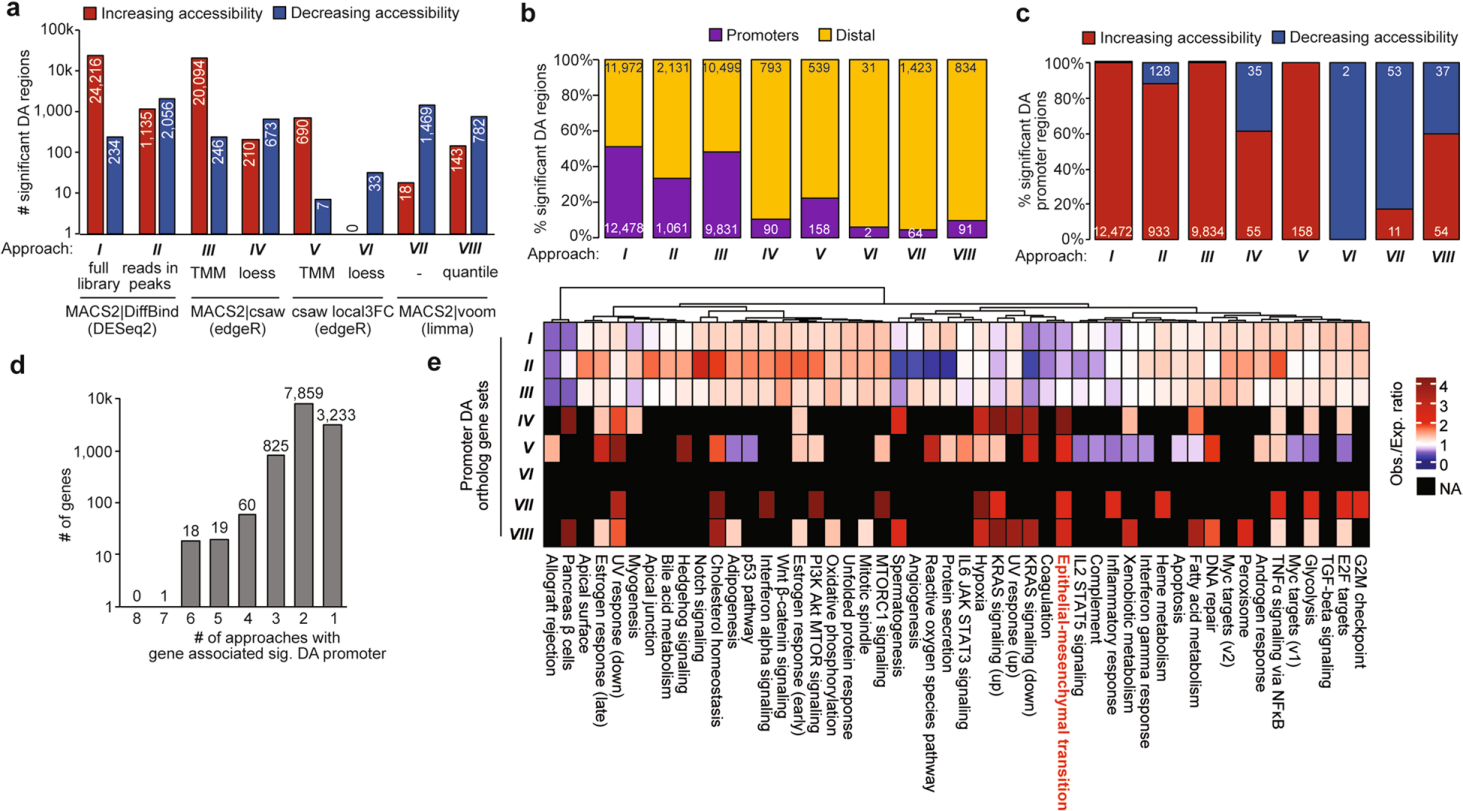
Output comparison of approaches for computing differential accessibility. **a** Output comparison of 8 approaches described in Fig. 1 for calling significant DA regions in ATAC-seq data, separated by increasing vs. decreasing accessibility regions. **b** Comparison of same 8 approaches divided by significant DA promoter regions (within 3 kb of a TSS) vs. distal (further than 3 kb of a TSS). **c** Comparison of significant DA promoter regions in all 8 approaches segregated by increasing vs. decreasing accessibility. **d** Quantification of overlapping genes associated with a significant DA promoter region between all 8 approaches. **e** Gene set enrichment of Hallmark MSigDB pathways among genes with DA promoters for all 8 approaches. Enrichment displayed as observed/expected ratio, where red values indicate pathway overrepresentation

We next performed a detailed comparative analysis of the DA outputs from all 8 different approaches. The number of significant DA regions identified by an FDR < 0.10 threshold ranged from 33 to 24,450 with varying proportions of regions increasing vs. decreasing accessibility (Fig. 2a). Genomic annotation of these peaks by *HOMER* [38] showed that gene promoters, defined as a region within 3 kb of a TSS, constituted varying extents of the DA regions ranging from 6% to 51% (Fig. 2b). However, in 6 out of the 8 tested approaches, gene promoters were predominantly increasing in accessibility overall (Fig. 2c). This comparative analysis permitted the probable conclusion that gene promoters are mostly increasing in accessibility in mutant cells, even though the global patterns observed in comparisons between each DA method show discordance.

Understanding what gene promoters display affected accessibility is biologically informative since promoter chromatin accessibility often correlates with transcription [7, 39]. We first asked how many genes were commonly identified as exhibiting a DA promoter region among the multiple tested approaches. Strikingly, no genes were found commonly among all 8 approaches, though certain sets of genes were commonly identified in multiple approaches (Fig. 2d). As shown by pathway enrichment analysis, choice of DA approach also led to differences in biological processes observed for promoters with affected chromatin accessibility. Epithelial–mesenchymal transition (EMT) was uniquely highlighted in significant DA promoter genes from DA approaches *IV*, *V*, *VII*, and *VIII* as opposed to other methods (Fig. 2e). We previously reported a role of *ARID1A* and *PIK3CA* mutations in EMT-related processes through multiple molecular and cell-based in vitro and in vivo assays [17].

A commonly used approach to validate chromatin accessibility observations is to compare the results with gene expression data. Next, we asked if those genes with a significant DA promoter region found in any one of the DA approaches were associated with differential expression (DE) in mutant vs. control endometrial epithelial populations. One issue is that quantifying direct overlap between promoter DA and gene DE is sensitive to the DA FDR threshold. To overcome this, we implemented a precision–recall (PR) curve to predict gene DE status (binary, based on RNA-seq FDR < 0.05 threshold) with promoter DA FDR values yielded by a given DA analysis. The PR curves showed modest predictive ability of promoter DA, though analyses *III*, *IV*, *V*, and *VIII* were better predictors of gene expression changes than the others (Additional file 1: Figure S1a). The predictive abilities are most apparent before 20% recall (i.e., 20% of all DE genes with a tested promoter ATAC region), indicating that gene expression changes observed in this experiment are not entirely determined by alterations in promoter chromatin accessibility.

We further investigated how FDR thresholding could affect DA outputs. We observed different FDR thresholds between our 8 different approaches that elicited a 5% null hypothesis rejection, ranging from FDR = 0.00331 (*I*) to FDR = 0.936 (*VI*) (Additional file 1: Figure S1b). As such, we compared the number of DE genes with a DA promoter region (Additional file 1: Figure S1c) and saw that this was highly dependent on FDR threshold. These results suggest that FDR thresholding can change between DA testing methods, and optimizing this aspect of the analysis may also improve results and interpretation. Collectively, all of these analyses underscore the importance of comparative analysis with multiple DA outputs before settling on conclusions. Furthermore, choosing a conservative normalization method may reduce both the need for such rigorous comparisons or use of multiple independent assays.

### Temporal chromatin accessibility measurements in yeast also display normalization bias

In addition to comparing the effects of genetic mutations or other treatment conditions, examining temporal changes in chromatin accessibility in cell populations is another application of ATAC-seq DA analysis. We utilized the Schep et al. [18] osmotic stress time-course ATAC-seq data set from yeast to determine if choice of DA analysis workflow yielded different results. Yeast cells were treated with 0.6 M NaCl and harvested cells for ATAC-seq at four, 15-min intervals, up to 60 min, for comparison against control cells at 0 min exposure (*n* = 2 per time point). An advantage to this data set is the inclusion of two control groups, one containing NaCl in the wash buffer and one without NaCl, which permits comparisons between two negative control groups. Schep et al. also utilized a published expression microarray data set by Ni et al. [40] from the same 0.6 M NaCl treatment design, in which three patterns (unchanged, upregulated, and downregulated) were defined based on osmotic stress response over time. Schep et al. reported that a subset of genes from each expression response pattern also displayed similar profiles with respect to promoter accessibility change [18].

Our workflow detected between 1271 and 1894 genome-wide naïve overlap peaks at any given time condition, resulting in between 2261 and 2601 tested DA regions depending on the DA analysis approach used. DA comparison of the two control groups with all 8 analytical approaches resulted in very few statistically significant DA regions (FDR < 0.05), ranging from 0 to 85 regions (Additional file 1: Figure S2). This in contrast with the 15-min (*n* = 2) vs. 0-min control (*n* = 4) comparison, in which between 491 and 1082 regions were determined significantly DA (Additional file 1: Figure S3a). Because the yeast genome is highly compact with functional genes and relatively few introns or other distal regulatory elements (compared to vertebrates), > 95% of DA regions at 15 min exposure were located within gene promoters (defined as − 2000 to + 200 bp from TSS) with every approach (Additional file 1: Figure S3b). We then determined the overlap of promoter regions displaying increasing or decreasing accessibility with each of the three gene expression patterns defined by Ni et al. We were able to classify DA regions and gene expression patterns for all 8 approaches, with up to 75% of the increasing accessibility promoter regions and up to 70% of the decreasing accessibility regions classified by expression changes (Additional file 1: Figure S3c). However, we noted a wide range in the number of genes displaying concordant gene expression and promoter chromatin accessibility changes (Additional file 1: Figure S3d).

**Fig. 3:**
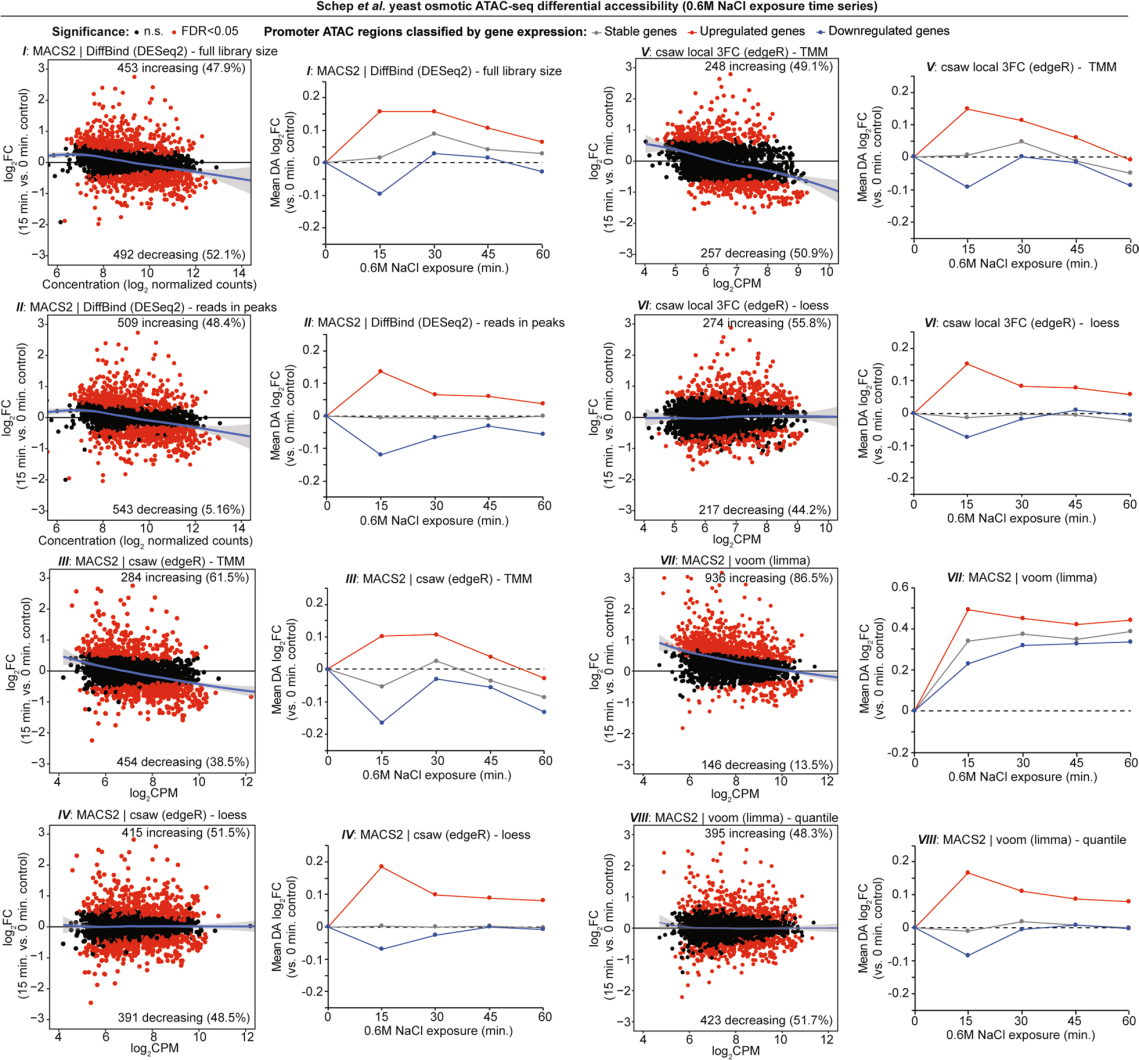
Comprehensive DA analysis and gene expression comparisons of yeast osmotic time-course series. Time series analysis of the Schep et al. osmotic stress in yeast ATAC-seq data set with all 8 DA approaches. MA plots are shown for 15-min exposure vs. 0-min controls and exemplary of global effects of data normalization. Time-course line plots depict the mean change in accessibility at each time point compared to control samples, for all gene promoter ATAC regions defined by respective gene expression changes. Gene expression changes following the same 0.6 M NaCl treatment reported by Ni et al. are defined as stable expression (gray line), upregulated expression (red line), and downregulated expression (blue line). See Additional file 1: Figure S4 for complete data and statistical analysis of time-course series with all 8 approaches

The most compelling observations occurred when we assessed the full spectrum of gene expression and chromatin accessibility changes across the entire time-course series. Certain DA analyses showed biologically expected changes in overall accessibility that reflected expression, such as *II*, *IV*, and *VIII*, while the accessibility profiles from other approaches appeared asymmetrical indicating technical bias (Fig. 3). With some DA analyses, strong DA statistical significance is observed among stably expressed genes, which are not expected to display accessibility changes (Additional file 1: Figure S4). In most cases, the MA plot profiles were predictive of the gene expression and chromatin accessibility patterns observed throughout the time course. In 4 DA methods with an asymmetrical MA plot trend (*I*, *III*, *V*, and *VII*), the displayed chromatin accessibility profiles over the time course did not match the direction of gene expression change (Fig. 3). Most importantly, even in a highly controlled time-course experiment in yeast, certain DA analyses can yield technically discordant results that do not align with the orthogonal assays.

### Generalized ATAC-seq workflow for differential chromatin accessibility analysis

Various studies have described ATAC-seq quality control and data processing for non-computational scientists, but few emphasize DA analysis [23–26]. Moreover, we noted a literature gap in the importance of standardizing molecular complexity before quantifying differences between experimental ATAC libraries. This, coupled with our observation that choice of normalization method impacts DA results, led us to develop a comprehensive and easy-to-follow computational workflow for differential ATAC-seq data analysis.

**Fig. 4:**
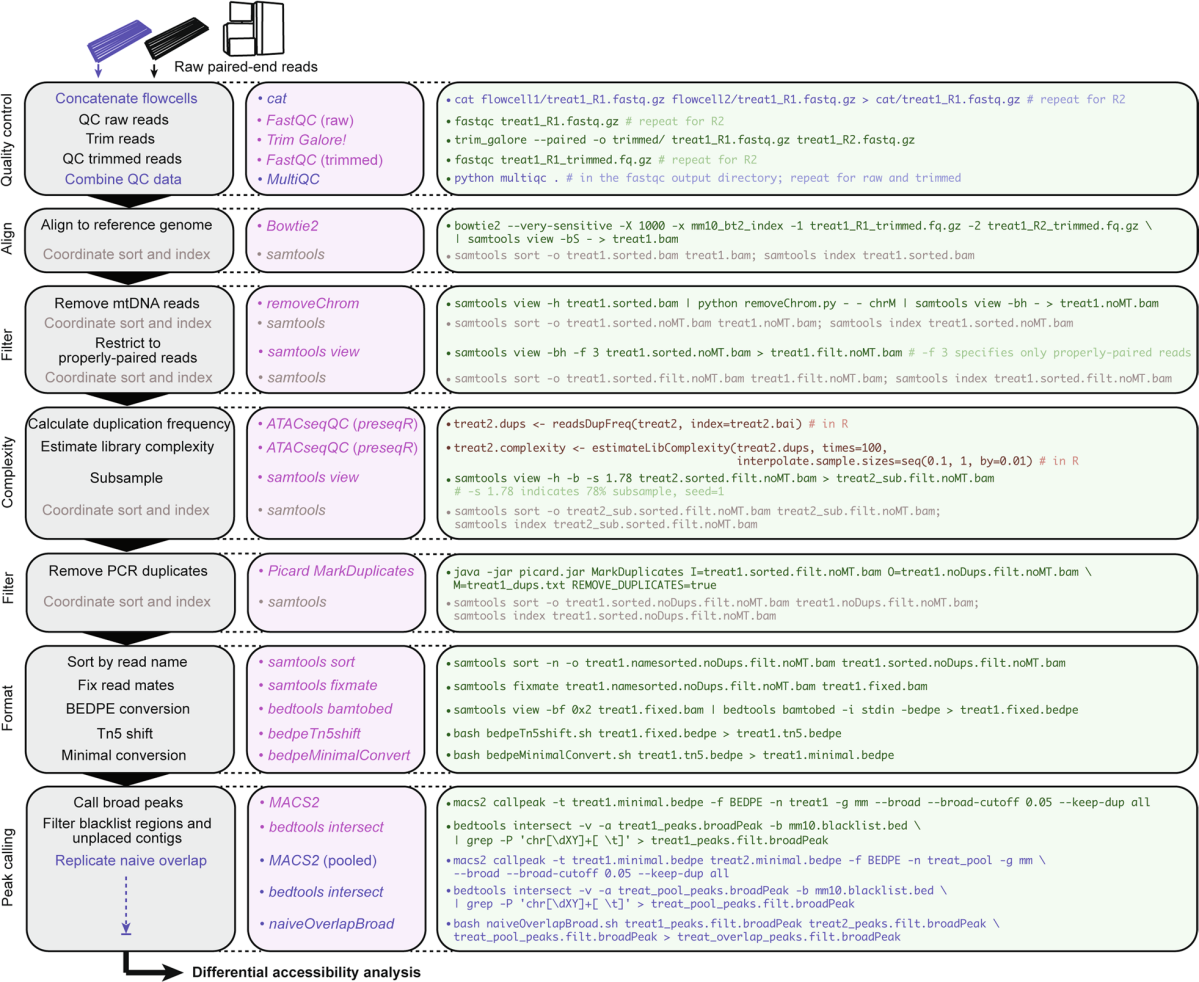
Generalized ATAC-seq data processing workflow intended for comparative analysis. Stepwise bioinformatics process and example commands for analyzing ATAC-seq data from raw reads to calling peaks for downstream differential accessibility analysis. Consider “treat1” as an example mouse ATAC-seq Illumina paired-end library. Blue text denotes optional or conditional steps dependent on experimental design and desired output. Users seeking only to discover replicate-concordant accessible regions in a singular cell state may wish to call naïve overlapping peaks, though this step is not necessary for differential accessibility analysis. Bash scripts for Tn5 coordinate shift (*bedpeTn5shift.sh*), minimal BEDPE format conversion (*bedpeMinimalConvert.sh*), and calling naïve overlap broad peaks (*naiveOverlapBroad.sh*) are located in the additional files section along with a machine-readable text version of this workflow

The workflow presented in Fig. 4 is devised to be widely applicable to any ATAC-seq data set or experimental design. It is based after the standardized ENCODE pipeline devised by Kundaje et al. (https://libraries.io/github/kundajelab/atac_dnase_pipelines) with modifications. Example applications include calling baseline accessible regions in naïve cells and identifying DA genomic regions. The field typically accepts data sets with at minimum two biological replicates for standard peak calling, as was established by ENCODE for ChIP-seq data [41], and at least two biological replicates are absolutely required for any DA statistical analysis. For each step, we have included a descriptive phrase along with the software tools used and example code. A detailed description of the workflow is available in Additional file 1: Methods, along with a machine-readable text version with comments (Additional file 2). Custom Unix scripts for certain workflow functions are also supplied (Additional files 3, 4, 5). Notably, we implemented the ENCODE-defined naïve overlap to determine biological replicate peak concordance. This method calls peaks on pooled replicates, and then identifies peaks displaying at least 50% overlap with all single replicate peaks. We have supplied a Unix shell script (*naiveOverlapBroad.sh*, Additional file 5) to execute this function for computing naïve overlap from two broadPeak replicates and can be easily modified to support more replicates. Additionally, publicly available blacklist regions (ENCODE consortium) refer to highly repetitive or unstructured regions that display artificially high signal in genomic experiments [42].

A major addition to our workflow is quantifying and normalizing library molecular complexity. Often times, it is desired to quantify and compare ATAC signal at different genomic loci which are not typically part of an ATAC-seq or differential ATAC-seq analysis framework. This could involve genomic tiling, i.e., quantifying signal in evenly distributed genomic intervals, or quantifying ATAC at regions defined through other assays, such as ChIP-seq, or *k*-means clustering [20, 43–45]. For certain analyses, it may not be appropriate to implement one of the normalization methods described herein, as determining proper biological or statistical assumptions may not be plausible. Rather, a linear transformation approach is often implemented, such as scaling libraries by read depth. However, this method assumes no differences in global library preparation biases, such as differing ATAC reaction efficiency. A potential result of such biases is affected sequence diversity which manifests in library molecular complexity, or the estimated number of sequenced molecules as determined by duplication rates. If uncorrected, libraries of equivalent read depth may be confounded by complexity in downstream analysis. Instead, libraries can be normalized to the estimated number of sequenced molecules, determined by duplication rates as the library molecular complexity. This method mitigates potential biases by considering that the relative distribution of transposase integration (reads at a given feature) is most biologically informative under an equivalent number of transposase reactions (total molecules sequenced, i.e., molecular complexity).

We suggest estimating the complexities of all samples in the compared conditions, and then performing a stochastic subsampling process in order to normalize all samples to equivalent molecular complexity. All analyses presented in this study have undergone this step unless specifically stated otherwise. The R packages *preseqR* and a wrapper *ATACseqQC* have implemented functions to estimate complexity by calculating a duplicate frequency matrix then estimating the number of unique molecules sequenced (i.e., molecular complexity) in each library sample [25, 46]. *samtools view* can then be used to subsample libraries based on these estimates. In support that the stochastic subsampling process should not greatly affect experimental results, a replicated analysis with two different random subsampling seeds yielded highly similar and overlapping results from peak calling (Additional file 1: Figure S5a) and DA analysis (Additional file 1: Figure S5b).

Among our sorted mouse epithelial cell ATAC-seq data set, control libraries had lower molecular complexities than mutant libraries (Additional file 1: Figure S6a, b), which we corrected by subsampling (Additional file 1: Figure S6c). By performing this complexity-normalization process, we had improved confidence that the observed ATAC differences were biological and not technical in nature. An example of the functional effects of complexity normalization is illustrated through ATAC signal quantification at a set of significant DA promoter regions (FDR < 0.10) defined by approach *IV*. 35 promoter regions calculated as significantly decreasing accessibility by this method did not yield statistical significance when quantifying ATAC RPKM in read depth-normalized libraries (Additional file 1: Figure S6d), but the decreasing accessibility patterns become more evident when we compare complexity-normalized libraries (*p *= 0.0196, two-tailed paired Wilcoxon test) (Additional file 1: Figure S6e). This analysis highlights the confounding effects of library molecular complexity in comparative ATAC-seq analysis. Moreover, it supports the use of complexity-normalized libraries for certain quantitative purposes in particular.

### Proposed workflow effectively retains ATAC-seq peak calls in an independent data set

**Fig. 5:**
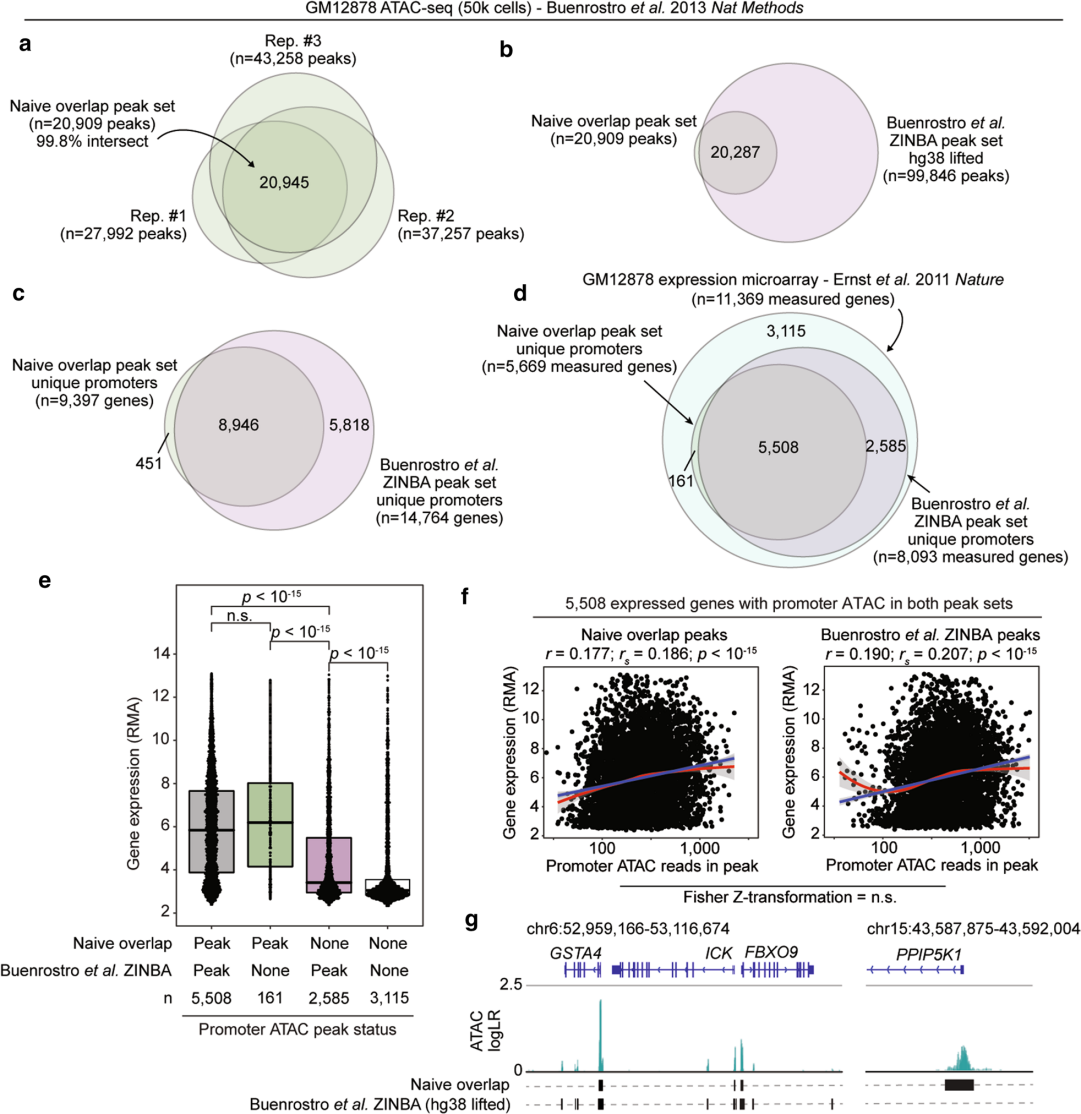
Conservative and relevant peak calling by proposed framework exemplified on Buenrostro et al. data. **a** Overlap of *MACS2* broad peaks called with proposed workflow between independent GM12878 ATAC-seq replicates from Buenrostro et al. Naïve overlap identifies 99.8% of fully replicate-intersecting peaks. **b** Genome-wide overlap of naïve overlap peak set generated herein compared to ZINBA peak set reported by Buenrostro et al. **c** Overlap of genes with detected ATAC promoters identified in the two peak sets as in **b**. **d** Overlap of expression-measured genes with detected ATAC promoters in the two peak sets compared to all measured genes. GM12878 expression data was pulled from a microarray data set generated by Ernst et al. **e** Microarray log_2_ expression levels (RMA) of genes segregated by promoter ATAC peak status detected between the two peak sets. Genes were binned as having a detected peak in both sets, only by naïve overlap herein, only by Buenrostro et al. ZINBA, or neither. Statistic is unpaired, two-tailed Wilcoxon test. **f** Correlation of promoter ATAC peak signal and gene expression for 5508 genes with a detected promoter ATAC peak in both peak sets. ATAC signal is quantified by reads in peak (log_10_ scale; linear values displayed on axis for clarity), and the strongest value was selected to represent promoters with multiple peaks. Correlation statistics displayed are Pearson and Spearman. Overlaid linear fit is displayed in red and loess in blue. Fisher Z-transformation was used to compare correlation coefficients between both peak sets. **g** Example ATAC-seq signal tracks showing peaks called (black bars) at different loci between the two peak sets. All three replicates are overlaid with darker colors representing overlapping replicates. *Y*-axis is log likelihood ratio of peak signal

To further assess the effectiveness of our ATAC-seq data analysis workflow, we tested it on one of the original reported data sets generated by Buenrostro et al. [8]. ATAC-seq libraries were generated on three replicates of 50,000 GM12878 human lymphoblastoid cells, and the reported bioinformatic analysis yielded a replicate-merged peak set of 99,885 accessible chromatin regions via *ZINBA* [47]. From these data, we are able to assay the ability for our proposed workflow to identify biologically relevant ATAC-seq peaks. Through our workflow, we identified 20,945 genomic regions which *MACS2* called a significant broad peak in all three replicates, of which 20,909 (99.8%) were also retained in the naïve overlap peak set indicating replicate peak region concordance > 50% (Fig. 5a). To directly compare hg38-aligned naïve overlap peaks called through our workflow with the hg19-aligned Buenrostro et al. *ZINBA* peak set, we lifted the hg19 coordinates to hg38 with 99.96% successful mapping rate [48]. We identified extremely strong concordance between the naïve overlap peak set and Buenrostro et al. *ZINBA* peak set, with over 97% of the naïve overlap peaks intersecting (Fig. 5b). This indicated that nearly all of the naïve overlap peak set regions were also identified with the Buenrostro et al. *ZINBA* peak set, but there were an additional nearly 80,000 peaks which were not identified in the naïve overlap peak set. When the two peak sets were annotated via *HOMER* [38], we observed even stronger concordance and conservation between the genes with an identified promoter ATAC-seq peak in both peak sets. Whereas only roughly 20% of the genome-wide Buenrostro et al. *ZINBA* peaks intersected with naïve overlap peaks, approximately 60% of the genes with an identified promoter ATAC-seq peak were also identified in the naïve overlap peak set (Fig. 5c).

By leveraging a GM12878 microarray gene expression data set from Ernst et al. [49], we were able to next compare the expression of genes which were identified as having a promoter ATAC-seq peak concordantly or uniquely between the two peak sets. Again, we observed strong concordance between the genes measured for expression by microarray which had a promoter ATAC-seq peak in the two peak sets (*p* = 0, hypergeometric enrichment), where 5508 were concordantly identified between both, 2585 were uniquely called in the Buenrostro et al. *ZINBA* peak set, and 161 were uniquely called in the naïve overlap peak set (Fig. 5d). We then compared the expression of the genes in each of these three bins. Genes were equivalently highly expressed that were concordantly identified as exhibiting a promoter ATAC between both peak sets or uniquely in the naïve overlap peak set, but the 2585 genes which were uniquely identified as having a promoter ATAC peak in the Buenrostro et al. *ZINBA* peak set were lowly expressed (Fig. 5e). Furthermore, all genes with a promoter ATAC peak in either of the peak sets were indeed overall more highly expressed than genes which never exhibited a promoter ATAC peak. A further extension of this analysis compared the correlation between promoter ATAC peak signal and respective gene expression for the 5508 commonly identified genes with microarray expression data, as it is widely accepted that higher promoter chromatin accessibility generally corresponds to higher gene expression. Correlations between promoter ATAC peak signal and gene expression were highly significant in both peak sets and analytical approaches and were not significantly different (Fig. 5f). We further observed that the naïve overlap peak set typically identified only conservative ATAC-seq peaks, yet there were also examples of robust ATAC signal which were only called as significant with the naïve overlap peak set, such as near the *PPIP5K1* promoter (Fig. 5g). Altogether, these analyses suggest that the workflow proposed herein is able to identify conservative regions of significant ATAC signal which corroborate gene expression observations.

### *csaw* differential accessibility workflow permits testing of multiple normalization methods

**Fig. 6:**
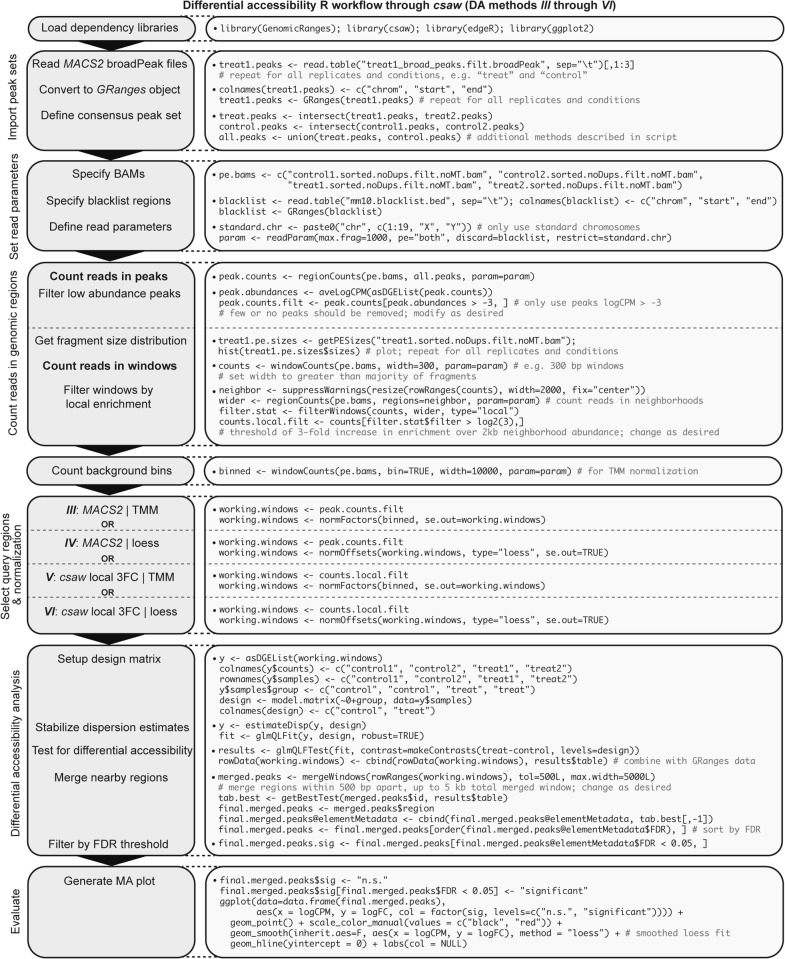
*csaw* workflow for multiple differential accessibility analyses in R. Graphical representation of proposed *csaw* workflow in R for calculating differential accessibility. Consider an experimental design with *n* = 2 biological replicates from two conditions: “treat” and “control”

We suggest using *csaw* as a go-to toolkit for standard downstream differential accessibility analysis. *csaw* is a flexible R package, originally designed for ChIP-seq analysis, which accepts sorted BAM files for DA quantification via *edgeR* quasi-likelihood methodology following any one of numerous implemented normalization methods to address many biological scenarios [28, 30]. Furthermore, *csaw* can be supplied *MACS2* peak coordinates for DA analysis or alternatively perform de novo ATAC enrichment detection with sliding windows and proper type I error control. These features make *csaw* an attractive tool for comprehensive DA analysis. For DA analysis, we have graphically represented a typical *csaw* workflow in R (Fig. 6), which is also available as a machine-readable text version (Additional file 6). This workflow outlines a continuation of Fig. 4 into complete DA analysis which aids users to compute DA approaches *III*, *IV*, *V*, and *VI* for output evaluation. The sensitivity of the proposed workflows in distinguishing signal from noise is further evident in DA analysis of two independent groups of negative controls from the Schep et al. yeast ATAC-seq data set (see Additional file 1: Figure S2).

The final steps of this graphic detail generation of an MA plot through *ggplot2* [50] to assess normalization outcomes. The distribution of an MA plot from DA analysis provides insight into trends or potential biases within the data, which users should critically consider. An upward or downward shift in the MA distribution could either indicate a global effect or substantial technical bias, and certain normalization methods will maintain or eliminate these features in the data. If the distribution further does not appear symmetrical along the horizontal axis, then a trended bias may be present which can be corrected with conservative normalization approaches like quantile or loess. Ultimately, the researcher must consider prior biological knowledge and the experimental design to determine if data trends should be accepted as biological or eliminated as technical.

## Discussion

This study has revealed that differential accessibility analysis of ATAC-seq data can be sensitive to underlying biases within the data, as might be expected. The design of the primary analyzed data set involved disrupting a chromatin remodeler subunit, which probably affects genome-scale chromatin structure, and comparing chromatin accessibility to that of control cells. Analytical interpretation is further confounded by in vivo heterogeneity in sorted cell populations. The consequence is that common tools and approaches for performing DA analysis give vastly different results that are difficult to interpret at first glance. MA plots of DA results displayed global ATAC distribution biases that were only thoroughly eliminated through certain normalization methods, like loess-based count adjustments. By comparing multiple DA analysis outputs, common patterns emerged that permitted high likelihood conclusions, such as gene promoters increasing in accessibility following chromatin remodeler subunit disruption. Application of precision–recall analysis to predict RNA-seq gene expression changes with DA data supported biological relevance of certain DA methods, since gene expression and promoter chromatin accessibility are known to correlate. This also further emphasized that pairing ATAC-seq with RNA-seq can be a useful approach to interpreting chromatin accessibility observations. FDR thresholding analyses suggest that optimizing the DA significance threshold can also improve results and interpretation. Overall, these results indicate that naively relying on one single DA analysis approach may lead to false conclusions, particularly so without assessing for presence of biases within the data. In the case of modifying or disrupting chromatin regulators, these biases may be commonplace and should be critically considered before further interpretation of data. Furthermore, certain methods are more conservative than others and can be initially selected to improve result confidence without the need to perform rigorous method comparisons.

The issue of intrinsic biases within ATAC-seq data for experimental designs where global changes may be expected are difficult to interpret. In the case of the experimental data presented here, limited prior knowledge is available whether or not disruption of this specific chromatin remodeler subunit should affect widespread chromatin structure, but it is not improbable. Thus, we were not able to determine if the inherent biases within the data are biological or technical, and we opted to remove them as technical. However, this decision could result in significant type II errors interpreted as technical in origin, if in fact they are truly related to the biology. Multiple downstream comparative analyses supported the loess normalization method as conservative and biologically relevant to our data set, so it was chosen as the normalization method with which to proceed forward for our published downstream analysis [17]. Though, significant information may have been lost by eliminating the global, trended biases that were observed within the data.

The observation that comparing ATAC-seq samples between two conditions may be subject to substantial experimental biases was actually accounted by Schep et al. in the yeast osmotic stress differential ATAC-seq report [18]. The authors specifically noted that, ‘variation in the degree of enrichment of fragments with open chromatin regions can affect differential accessibility measurements between ATAC-seq samples’. As such, they followed count quantile normalization with a lowess curve fit transformation to eliminate trended biases within the data. This account further supports that differential ATAC-seq analyses are sensitive to experimental, technical biases, such as ATAC reaction efficiency, as well as the rationale behind use of the loess normalization method to elicit a highly conservative DA result. Still, this normalization method is not widely used for ATAC-seq DA analysis. It is also important to note that similar observations have been reported for ChIP-seq analysis, where non-linear loess normalization methods were proposed and developed to eliminate systematic errors between libraries [51]. However, the rationale behind a loess fit assumes that the data should be symmetrical without a global change observed, so users should be aware that implementing this technique may hide any true global alterations present between the two conditions.

Biases inherent to quantitative genomic techniques based on chromatin feature signal enrichment have been observed and considered previously [52]. ATAC-seq fundamentally relies on an enzymatic reaction for library construction, which is likely to be affected by amount of enzyme, number of nuclei, and chromatin compaction and structure. ATAC-seq was recently reported to exhibit a sequence-specific bias distinct from DNase I libraries [53]. MNase digestion has previously been shown to be highly sensitive to enzymatic activity and also displays sequence specificity bias [54, 55]. In ChIP-seq libraries, potential bias in factor binding measurements is thought to be derived from local transcriptional activity and chromatin structural properties [56]. Our current investigation has shown that quantitative comparative analysis of ATAC libraries is confounded by technical bias. When alternative methods to detect chromatin accessibility changes are unavailable (e.g., due to low cell numbers or input retrieval), users should empirically determine the most appropriate normalization methods and employ orthogonal assays, such as gene expression, for comparisons with ATAC-seq data.

Calculating linear library normalization factors is a standard approach, but sensitive to biases. Here, we have shown that MA plots are a simple, qualitative approach to identifying systematic biases in experimental ATAC-seq libraries, as others have shown with ChIP-seq data [57]. Calculating the fraction of reads in peaks (FRiP) score, as described by ENCODE [41] and streamlined by *DiffBind*, is a simple method to evaluating ATAC efficiency between libraries to determine whether or not a systematic bias may be present. In the case of substantially differing ATAC efficiencies, linear normalization factors can be derived from only reads in peaks, as in *DiffBind*, or by applying TMM to only high abundance regions, as is suggested by the authors of *csaw*. As we have discussed, *csaw* also has a non-linear loess-based count normalization which can be easily implemented to assess its effects on DA calculation after the above considerations.

Before DA analysis, the most significant addition to our proposed standard ATAC-seq data analysis workflow is normalization of library complexity by random subsampling. In the case that ATAC reaction efficiencies are different between libraries, it is advised to investigate the library molecular complexity as a technical source for this error arising during sequencing. If less input material is retrieved from transposition for certain samples, and more PCR amplification cycles are required as a result [58], then bias is introduced into the amplified fragments dependent on GC content, fragment length, and oligonucleotide complexity [59]. If libraries are complexity-normalized within an experimental design to the same estimated number of unique molecules, then direct quantitative comparison of unique fragments between conditions is more informative, e.g., integer feature read counts at loci identified in other assays. Like the systematic biases present in DA analysis, however, library complexity normalization is currently also a flawed concept. In the case that a drastic global decrease in chromatin accessibility is truly biological, then less transposed DNA fragment retrieval is expected, and these libraries might exhibit lower complexity. In this scenario, complexity normalization may not be desired as it would confound the true chromatin biology. However, without independent knowledge, this decision is not easily made. Notably, others have approached similar problems in ChIP-seq through addition of exogenous chromatin from a distinct species as reference for IP efficiency and sequencing bias, referred to as “spike-in” controls [60, 61]. More recently, this technique has been extended to incorporating a fraction of spike-in live cells prior to lysis in the ATAC-seq protocol [62], and the effects of spike-in normalization of ATAC-seq data could help establish technical or biological basis for global accessibility patterns, in principle.

The presented ATAC-seq workflow and the suggested DA toolkit are not absolute and should be improved as analytical methods continue to emerge. For example, one newly developed method implementing a hidden Markov model (HMM) showed better performance for differential ChIP-seq analysis than *csaw* or other sliding window approaches, which suffer in identifying narrow changes within large genomic domains [63]. At least one HMM tool has been developed specifically for calling nucleosome-free regions within ATAC-seq data, and its implementation could be extended to differential analysis [64]. While our framework was able to identify both broad and narrow regions of strong ATAC signal in the Buenrostro et al. GM12878 data set, the peak calling thresholds may be too strict to identify truly nucleosome-depleted regions displaying weak signal. Methods also currently exist for correcting sequence-specific biases resulting from various chromatin digestion and enrichment techniques, and the extent of analytical affect from this correction should be evaluated [65, 66]. Currently, ATAC-seq normalization and DA approaches should be carefully considered to appropriately reduce the inherent biases within each analysis.

## Conclusions

We present data indicating that ATAC-seq is sensitive to bias when comparing chromatin accessibility across multiple conditions. We compared several commonly used, published methods for calculating differential accessibility to our previously reported in vivo ATAC-seq data set as well as a yeast ATAC-seq time series data set, and we observe conflicting results dependent upon the normalization method used. We provide intuitive, standardized bioinformatics methodology for analyzing ATAC-seq data by non-computational scientists. Our validated workflow also includes a critical, complexity-normalization step. Altogether, we argue that researchers should properly normalize ATAC-seq data before calculating differential accessibility.

## Methods

### Analyzed data sets

Sequencing data used for analyses presented in this manuscript were downloaded from GEO accessions GSE121198, GSE66386, GSE47753, and GSE26312. Yeast genes with distinct expression response patterns following 0.6 M NaCl exposure were defined and extracted from Supp. Table 4 reported by Ni et al. [40]

### ATAC-seq and differential accessibility analysis

See Figs. 4 and 6, and Additional file 1: Method for complete workflow details and description. Mouse libraries were aligned to mm10 genome assembly, and yeast libraries were aligned to sacCer3 genome assembly. ATAC-seq peaks were not filtered for blacklisted regions in yeast, as they are not defined in this organism. Presented DA analyses were computed through the use of R packages *DiffBind*, *DESeq2, csaw*, *edgeR*, *voom*, and *limma* as described in the “Results” section [28–33]. Workflows for all tools are described in detail in Additional file 1: Methods. The BAM files supplied to DA tools correspond to the coordinate sorted/indexed, duplicate removed, complexity-normalized, properly paired restricted, non-mitochondrial, paired-end BAM files generated as described in Fig. 4.

### RNA-seq analysis

Differential gene expression results from previously reported RNA-seq data of *LtfCre*^*0/*+^*; (Gt)R26*^Pik3ca*H1047R^*; Arid1a*^*fl/fl*^ vs. control sorted mouse endometrial epithelial cells were extracted from GEO accession GSE129784. 3481 significant DE genes were selected by FDR < 0.05 filtering. 24,097 total expressed genes were used as gene universe for enrichment analyses.

### GM12878 gene expression microarray analysis

Raw data were downloaded from GEO for both GM12878 replicates generated by Affymetrix HT Human Genome U133A Array. CEL files were read into R through the *affy* package and normalized via the Robust Multi-Array Average (RMA) expression measure. RMA values are reported as log_2_ scale. The mean RMA value of both replicates was used for analyses in this manuscript. The 22,277 measured probes were collapsed to 11,369 genes with a unique Ensembl and symbol identifier [67]. Expression comparisons of gene groups binned by ATAC peak status was achieved by unpaired, two-tailed Wilcoxon test.

### Bioinformatics and statistics

Mouse and human ATAC-seq peak coordinates were annotated by *HOMER* [38] with a modification to *cis*-promoter classification as within 3000 bp of a canonical gene TSS. Yeast genomic regions were annotated by *TxDb.Scerevisiae.UCSC.sacCer3.sgdGene* R package using the *genes()* and *promoters()* functions with default settings (yeast promoters are defined as − 2000 to + 200 bp around TSS) [68]. Unweighted precision–recall curves were generated by the *PRROC* R package using the *pr.curve()* function [69]. For PR curve predictive analysis, the strongest (lowest) promoter DA FDR value was selected for each expressed gene, for each approach, and this value was used to predict boolean DE gene status segregated by RNA-seq DGE *DESeq2* FDR < 0.05 threshold. Read counts in ATAC-seq peaks were calculated by *HOMER* for correlation and box dot plot quantification. *MACS2* was used to generate ATAC-seq signal tracks for display in *IGV* [27, 70]. MSigDB Hallmark pathway enrichment was reported as observed/expected ratios derived from expressed gene sets compared to the respective expressed gene universe [71]. Pathway hierarchical clustering by Euclidean distance and heatmap were generated by *ComplexHeatmap* [72]. *biomaRt* was used for all gene nomenclature and mouse–human ortholog conversions [73]. The cumulative hypergeometric distribution was calculated in R for enrichment tests. *ggplot2* was used for certain plotting applications throughout this manuscript [50].

## Supplementary information

**Additional file 1.** Supplementary Information (Methods and Figures).

**Additional file 2.***ATACseq_workflow.txt*—Example machine-readable Fig. 4 workflow including stepwise unix and R commands for ATAC-seq data processing.

**Additional file 3.** *bedpeTn5shift.sh*—Bash script for shifting coordinates in standard 10-column format BEDPE files to compensate for Tn5 adapter insertion as described in Buenrostro et al. See Fig. 4 for usage.

**Additional file 4.***bedpeMinimalConvert.sh*—Bash script for converting standard 10-column format BEDPE to the “minimal” format defined by *MACS2*. See Fig. 4 for usage.

**Additional file 5.***naiveOverlapBroad.sh*—Bash script for calculating naïve overlap broad peak set from 2 individual replicate peak sets and a pooled replicate peak set. Can be modified for to accept more replicates as desired. See Fig. 4 for usage.

**Additional file 6. ***csaw_workflow.R*—Example R workflow for differential accessibility analysis with *csaw* as graphically displayed in Fig. 6. Describes process for both TMM and loess normalizations and either supplying *MACS2* peak sets as query regions or identifying de novo locally enriched windows.

## Data Availability

All analyzed data sets are publicly available at GEO accessions GSE121198, GSE66386, GSE47753, and GSE26312. Yeast genes with distinct expression response patterns following 0.6 M NaCl exposure were defined and extracted from Supp. Table 4 reported by Ni et al. [73]. All workflow and custom function scripts are available in the “Additional files” section as well as a GitHub repository (https://github.com/reskejak/ATAC-seq). A detailed description of the workflow commands in Figs. 4 and 6 as well as all different DA analysis methods are available in Additional file 1: Methods.

## Abbreviations

ATAC: Assay for Transposase-Accessible Chromatin
DA: Differential accessibility; differentially accessible
DE: Differential expression; differentially expressed
EMT: Epithelial–mesenchymal transition
FRiP: Fraction of reads in peaks
GLM: Generalized linear model
HMM: Hidden Markov model
loess: Locally estimated scatterplot smoothing
log_2_CPM: Log_2_ counts per million; logCPM
log_2_FC: Log_2_ fold change; logFC
lowess: Locally weighted scatterplot smoothing
MNase: Micrococcal nuclease
PR: Precision–recall
RPK: Reads per kilobase
RPKM: Reads per kilobase per million mapped reads
TMM: Trimmed mean of *M* values
