# Supplementary material for: ATAC-seq normalization method can significantly affect differential accessibility analysis and interpretation
Source: Epigenetics Chromatin. 2020 Apr 22;13:22. doi: 10.1186/s13072-020-00342-y (PMC7178746; doi:10.1186/s13072-020-00342-y)
Supplement: Supplementary file 1 — Additional file 1. Supplementary Information (Methods and Figures). [file 13072_2020_342_MOESM1_ESM.pdf]

ATAC-seq normalization method can significantly affect differential accessibility analysis and interpretation

Reske *et al.*

Additional File 1: Supplementary Information

Supplementary Methods

ATAC-seq analysis. This section provides detailed explanation of the Figure 4 workflow graphic. Throughout the workflow, steps should be performed on all replicates and conditions in parallel. A machine-readable text version of this workflow with further comments is supplied in the additional files section. Custom unix scripts for certain workflow functions are also supplied in the additional files section. Scripts are additionally available at the following GitHub repository: https://github.com/reskejak/ATAC-seq. Note that this workflow was originally written for mouse analysis and certain steps should be changed as necessary for application to other organisms.

In brief, the workflow begins with concatenation of any library read technical replicates, if necessary or applicable. Reads should then be trimmed and analyzed for quality control measures prior to genome alignment via *Bowtie2*[1]. To avoid issues with downstream filtering commands or manipulation, it is good practice to coordinate sort and index BAM intermediates after each step forward via *samtools*[2]. Reads mapping to the mitochondrial genome are then removed from the aligned BAM, such as with the *removeChrom* python script developed by Harvard Informatics (https://github.com/harvardinformatics/ATAC-seq). Mitochondrial read contamination is a well-documented issue with ATAC libraries due to lack of histones in mtDNA, and improved protocols have gone as far as including additional detergents to reduce contamination[3]. A further filtering step then retains only properly-paired reads for downstream use.

At this point, we suggest estimating the complexities of all samples in the compared conditions, and then performing a stochastic subsampling process in order to standardize all samples to equivalent molecular complexity. The R packages *preseqR* and a wrapper *ATACseqQC* have implemented functions to estimate complexity by calculating a duplicate frequency matrix

then estimating the number of unique molecules sequenced in each library sample[4,5]. *samtools view* can then be used to subsample libraries based on these estimates.

After standardizing library complexity across the experimental design, remove PCR duplicates e.g. through *Picard MarkDuplicates* (http://broadinstitute.github.io/picard/). The next steps involve converting the BAMs into paired-end BED format (BEDPE) for downstream peak calling. Firstly, sort the duplicate-removed BAMs by read names and fix associated read mate information. Converting to BEDPE is then achieved via *bedtools*[6]. In this format, a 9 bp coordinate shift can now be carried out to compensate for Tn5 transposase adapter insertion, which is practically achieved by a +4 and -5 bp shift to the Watson and Crick strand coordinates, respectively. The Tn5 shift should only minorly affect peak calling but is likely more important for high-resolution mapping such as motif footprinting. This step is largely a historic formality as was first reported by Buenrostro *et al.*[7], and we have included a bash script (*bedpeTn5shift.sh*) that will perform this adjustment via *awk*. Finally, *MACS2* requires a minimal 4-column BEDPE format that is collapsed from the standard 10-column *bedtools* format. We have also included a bash script (*bedpeMinimalConvert.sh*) to perform this minimal BEDPE format conversion.

Significant broad peaks can then be called from the minimal BEDPE individual replicates via *MACS2* without a supplied input sample[8]. Peaks should then be filtered with *bedtools* to remove low mappability, highly repetitive "blacklisted" genomic regions, which were previously identified in a comprehensive analysis of ENCODE data[9]. Removing peaks mapped to unplaced chromosome contigs is also suggested. At this stage in the workflow, one could proceed directly into our suggested DA analysis method with the individual replicate peak sets. However, it is often desirable to identify regions which consistently display ATAC peaks in all replicates for a given condition. For this purpose, we implemented the ENCODE-defined naïve overlap to determine

biological replicate peak concordance. This method calls peaks on pooled replicates, and then identifies peaks displaying at least 50% overlap with all single replicate peaks. We have supplied a bash script (*naiveOverlapBroad.sh*) to execute this function for computing naïve overlap from two broadPeak replicates, and it may be easily modified to support more replicates.

Differential accessibility analysis. Workflows for all implemented DA tools will be described in detail in this section. The *csaw* portion of this section describes the Figure 6 workflow, for which a machine-readable R script is available in the additional files section as well as in the following GitHub repository: https://github.com/reskejak/ATAC-seq. The BAM files supplied to DA tools correspond to the coordinate sorted/indexed, duplicate removed, complexity normalized, properly-paired restricted, non-mitochondrial, paired-end BAM files generated as described in the previous ATAC-seq analysis section, the Figure 4 workflow graphic, and further by Wilson & Reske *et al*[10].

For *DiffBind*[11], an experimental design sample table can be generated in R or a text editor in the format as described in the manual. This includes the columns "ID", "Factor", "Condition", "Replicate", "bamReads", "Peaks", and "PeakCaller". The field "bamControl" is not included for ATAC-seq analysis. The experiment DBA object is constructed through *dba()* using this "sampleSheet" table, and *DESeq2*[12] analysis is specified via \`AnalysisMethod=DBA_DESEQ2\` with option \`minOverlap=2\`. Average fragment size parameter was supplied as a list of all replicates using the values obtained from each *MACS2* .xls output file. *dba.count()* is then used on the experiment DBA object to count reads in peaks, followed by *dba.contrast()* with parameters \`minMembers=2\` and \`categories=DBA_FACTOR\`. The DBA object will then construct a consensus peak matrix for further DA interrogation. DA is then calculated via *dba.analyze()*, where the two normalization methods reported correspond to those with the Boolean operator \`bFullLibrarySize\`. A scalar count normalization by sample library total read depth is achieved

with `bFullLibrarySize=TRUE`, whereas `bFullLibrarySize=FALSE` will only use reads that are located within the consensus peak matrix for scalar normalization. *dba.report()* then outputs the DA results for only significant regions by default (FDR < 0.05), or the parameter `th=1` can be used to elicit results for all regions tested within the consensus peak matrix with their associated statistics.

For *csaw*[13], DA can be computed by either supplying a pre-defined peak set such as from *MACS2* or by calling enriched regions *de novo* through the implemented sliding window method. Both approaches will be outlined here. When starting with a pre-defined peak set, firstly import peak set BED files and construct *GRanges*[14] objects, then define a consensus peak set desired for further interrogation. For example, the consensus peak set, p, could be derived from 1) the union of all replicate peak sets for both condition, $p = (\bigcup_{j=1}^{n} e_j) \cup (\bigcup_{j=1}^{n} c_j)$, 2) the union of only naïve overlap peaks for both conditions, $p = e_{NOP} \cup c_{NOP}$, or 3) the union of condition peaks with any partial intersect for all replicates in a given condition, $p = (\bigcap_{j=1}^{n} e_j) \cup (\bigcap_{j=1}^{n} c_j)$, where e_1, …, e_n are replicate peak sets for the experimental condition, c_1, …, c_n are replicate peak sets for the control condition, e_{NOP} is the naïve overlap peak set for the experimental condition, and c_{NOP} is the naïve overlap peak set for the control condition. The latter of which (3) was selected for the presented analysis and is implemented in Figure 6. Read parameters should then be defined through *readParam()* specifying paired-end data and option `max.frag=1000`, to remove fragments over 1 kilobase in concordance with the library size-selection step. The `discard` parameter should be supplied with the blacklisted regions described earlier and `restrict` specified to standard chromosomes. Then, count reads in the specified consensus peak set windows by *regionCounts()*, and subsequently filter low abundance peaks e.g. by a logCPM > -3 threshold as used in this analysis. For normalization, TMM firstly requires counting of large background bins

through *windowCounts()* with `bin=TRUE`, and a standard parameter here is `width=10000` for 10 kb bins. Then, *normFactors()* will generate TMM[15] scaling factors based on the background binned counts. If instead desired, the loess-based normalization can be issued through *normOffsets()* with parameters `type="loess"` and `se.out=TRUE`, thereby writing the log-based offsets to the peak count matrix. Then, for DA analysis through *edgeR*[16], build a design model matrix, stabilize estimates with empirical bayes function *estimateDisp()*, fit the quasi-likelihood negative binomial model with *glmQLFit()*, specify contrast with *makeContrasts()*, and compute the quasi-likelihood F-tests with *glmQLFTest()*. Reference the *edgeR* manual for more information on constructing the design matrix. Finally, merge proximal tested windows by *mergeWindows()*, where a typical analysis will merge windows up to 500 bp apart for a maximal merged window size of 5 kb, as was performed in Figure 6 analysis. Then, use the most significant window as a statistical representation of the merged window with *getBestTest()*. The final window set can be filtered by a desired FDR threshold to determine significant DA regions.

If instead it is desired to identify *de novo* locally-enriched windows in *csaw* without prior peak calling, firstly assess the fragment length distribution with *getPEsizes()* to select an optimal window size. The window size is a critical parameter and should be set to larger than the majority of fragments; see *csaw* manual for more details. 300 bp was the optimal window size selected for the data analyzed here, so read windows were counted throughout the genome via *windowCounts()* with `width=300`. Next, there are numerous ways to filter uninteresting windows, one of which being local enrichment. We used a 2 kilobase neighborhood local background estimator to filter for windows only with a 3-fold increase in enrichment over neighborhood abundance. This was achieved by widening windows with *resize()*, counting neighborhood reads with *regionCounts()*, and filtering low enrichment windows with *filterWindows()*. Then, the locally enriched windows

can be subject to DA analysis, as described above, by implementing a normalization method, building a model, stabilizing estimates, fitting the model, and so forth. The supplied *csaw* workflow R script details commands for the entire DA process described here for both TMM and loess normalization and either using a prior defined *MACS2* peak set or identifying *de novo* locally-enriched windows.

For *voom*[17] methods (*VII* and *VIII*), analyses presented in the manuscript were conducted by first reading *MACS2* peak sets into *csaw* for counting and filtering as described above, though one could also apply *voom* methods to *csaw de novo* locally-enriched windows as well. The window counts table was extracted from *csaw* by *assay()* and converted into a data frame for further manipulation. After setting up the model matrix and contrasts, normalization and mean-variance estimation was computed by *voom()*. Quantile normalization was applied with the *voom* option `normalize.method="quantile"`, which applies the Bolstad *et al.* quantile normalization method that has also been used for ATAC-seq by other groups[18,19]. Through *limma*[20], a linear model was then fit with *lmFit()*, followed by *contrasts.fit()*, and *eBayes()* for moderated statistics and hypothesis testing. *topTable()* was used to extract full DA results with option `n=Inf`. See *limma* manual for more details on model matrix and contrast design.

References

1. Langmead, B. & Salzberg, S.L. Fast gapped-read alignment with Bowtie 2. *Nat Methods* **9**, 357-9 (2012).
2. Li, H. *et al.* The Sequence Alignment/Map format and SAMtools. *Bioinformatics* **25**, 2078-9 (2009).
3. Corces, M.R. *et al.* An improved ATAC-seq protocol reduces background and enables interrogation of frozen tissues. *Nat Methods* **14**, 959-962 (2017).

4. Ou, J. *et al.* ATACseqQC: a Bioconductor package for post-alignment quality assessment of ATAC-seq data. *BMC Genomics* **19**, 169 (2018).

5. Daley, T. & Smith, A.D. Predicting the molecular complexity of sequencing libraries. *Nat Methods* **10**, 325-7 (2013).

6. Quinlan, A.R. & Hall, I.M. BEDTools: a flexible suite of utilities for comparing genomic features. *Bioinformatics* **26**, 841-2 (2010).

7. Buenrostro, J.D., Giresi, P.G., Zaba, L.C., Chang, H.Y. & Greenleaf, W.J. Transposition of native chromatin for fast and sensitive epigenomic profiling of open chromatin, DNA-binding proteins and nucleosome position. *Nat Methods* **10**, 1213-8 (2013).

8. Zhang, Y. *et al.* Model-based analysis of ChIP-Seq (MACS). *Genome Biol* **9**, R137 (2008).

9. Amemiya, H.M., Kundaje, A. & Boyle, A.P. The ENCODE Blacklist: Identification of Problematic Regions of the Genome. *Sci Rep* **9**, 9354 (2019).

10. Wilson, M.R. *et al.* ARID1A and PI3-kinase pathway mutations in the endometrium drive epithelial transdifferentiation and collective invasion. *Nat Commun* **10**, 3554 (2019).

11. Stark, R. & Brown, G. DiffBind: differential binding analysis of ChIP-seq peak data. (2011).

12. Love, M.I., Huber, W. & Anders, S. Moderated estimation of fold change and dispersion for RNA-seq data with DESeq2. *Genome Biol* **15**, 550 (2014).

13. Lun, A.T. & Smyth, G.K. csaw: a Bioconductor package for differential binding analysis of ChIP-seq data using sliding windows. *Nucleic Acids Res* **44**, e45 (2016).

14. Lawrence, M. *et al.* Software for computing and annotating genomic ranges. *PLoS Comput Biol* **9**, e1003118 (2013).

15. Robinson, M.D. & Oshlack, A. A scaling normalization method for differential expression analysis of RNA-seq data. *Genome Biol* **11**, R25 (2010).

16. Robinson, M.D., McCarthy, D.J. & Smyth, G.K. edgeR: a Bioconductor package for differential expression analysis of digital gene expression data. *Bioinformatics* **26**, 139-40 (2010).

17. Law, C.W., Chen, Y., Shi, W. & Smyth, G.K. voom: Precision weights unlock linear model analysis tools for RNA-seq read counts. *Genome Biol* **15**, R29 (2014).

18. Bolstad, B.M., Irizarry, R.A., Astrand, M. & Speed, T.P. A comparison of normalization methods for high density oligonucleotide array data based on variance and bias. *Bioinformatics* **19**, 185-93 (2003).

19. Corces, M.R. *et al.* The chromatin accessibility landscape of primary human cancers. *Science* **362**(2018).

20. Ritchie, M.E. *et al.* limma powers differential expression analyses for RNA-sequencing and microarray studies. *Nucleic Acids Res* **43**, e47 (2015).

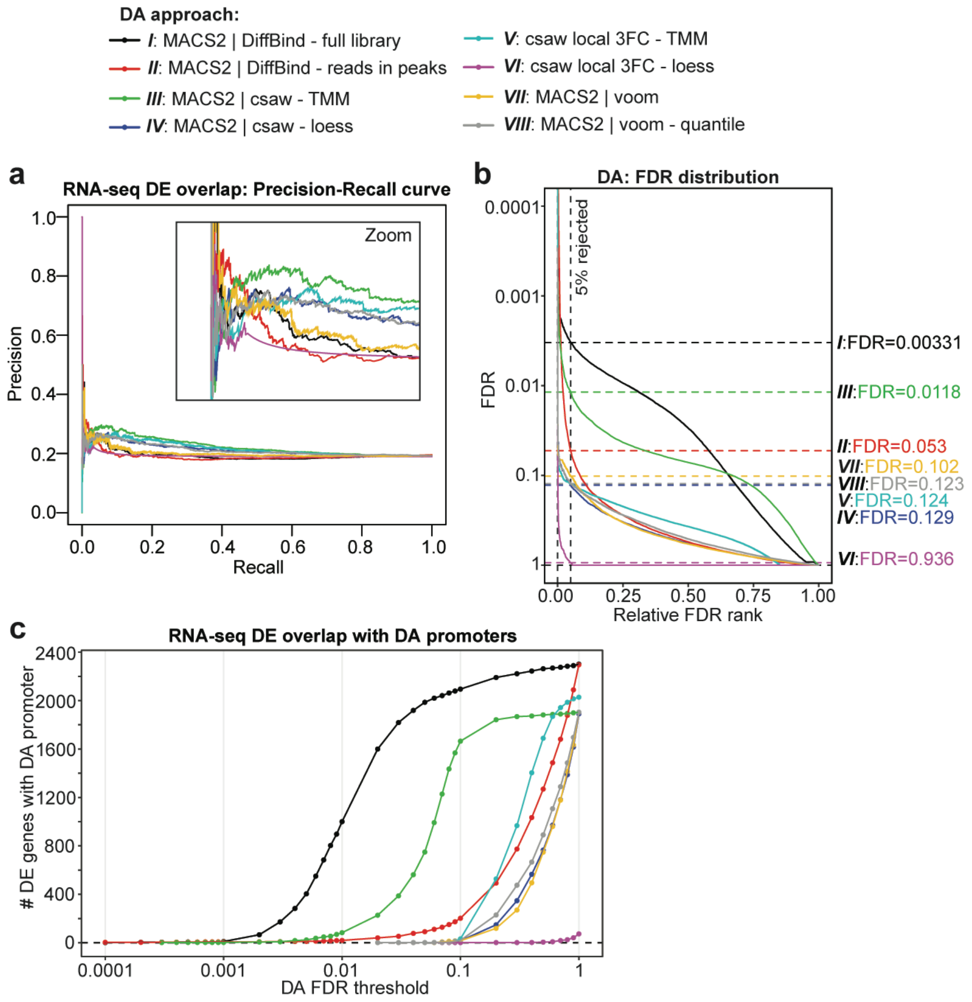

Fig. S1 | Differential gene expression overlap and FDR thresholding analyses. Color legend for the 8 DA approaches is located in the upper-right panel and applies to the entire figure. **a**, Precision-Recall curve (higher is better) predicting RNA-seq gene differential expression (DE) by promoter DA FDR value. Zoom inset depicts differences in approach specificity at low recall. Overall AUC values are similar for all curves. **b**, distribution of DA FDR values for all 8 approaches. Horizontal lines depict the approach-specific FDR thresholds meeting a 5% hypothesis rejection rate (vertical line). **c**, FDR thresholding analysis of number of RNA-seq DE genes overlapping with DA promoters.

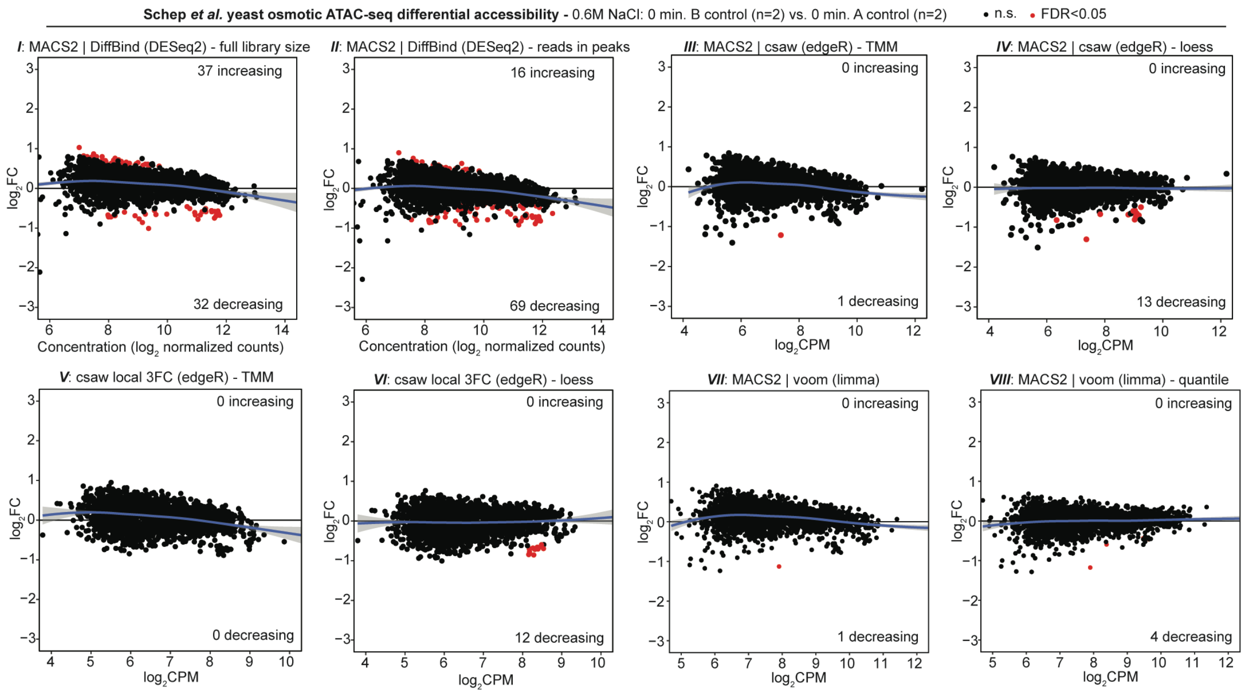

Fig. S2 | Negative control DA comparison of two control groups from Schep *et al.* yeast data set. MA plots from all 8 DA methods applied to a negative control comparison of two 0 minute control groups (n=2 each) from the Schep et al. yeast osmotic stress ATAC-seq data set.

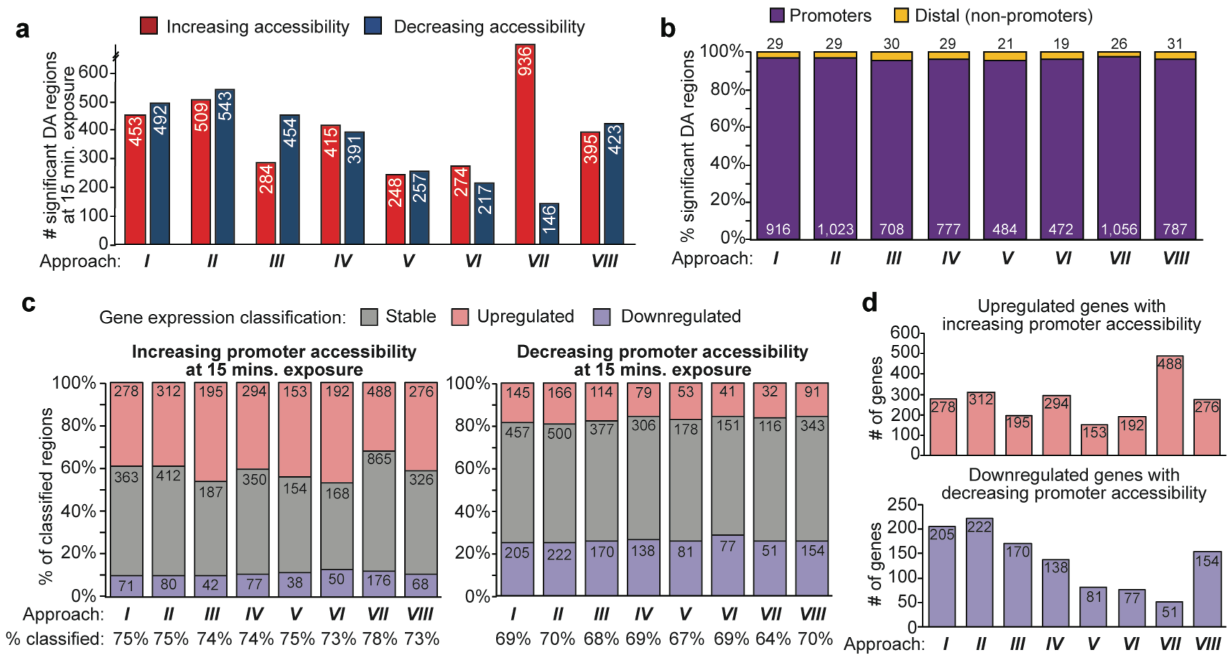

Fig. S3 | Extended analysis of DA methods on Schep *et al.* yeast osmotic stress ATAC-seq time course series. a, Genome-wide significant DA regions (FDR < 0.05) calculated by each of the 8 DA analyses at 15 minutes exposure vs. 0 minute control, separated by increasing vs. decreasing accessibility change. **b**, Breakdown of promoter (-2000 to +200 bp around TSS) vs. distal (non-promoter) annotation of significant DA regions from each of the 8 DA analyses again at 15 minutes exposure. **c**, Classification of significantly increasing (left) vs. decreasing (right) accessibility promoter regions at 15 minutes exposure based on Ni *et al.* gene expression response to the same osmotic stress conditions. **d**, Integer number of genes displaying concordant expression response and promoter accessibility changes at 15 minutes exposure, classified as in **c**.

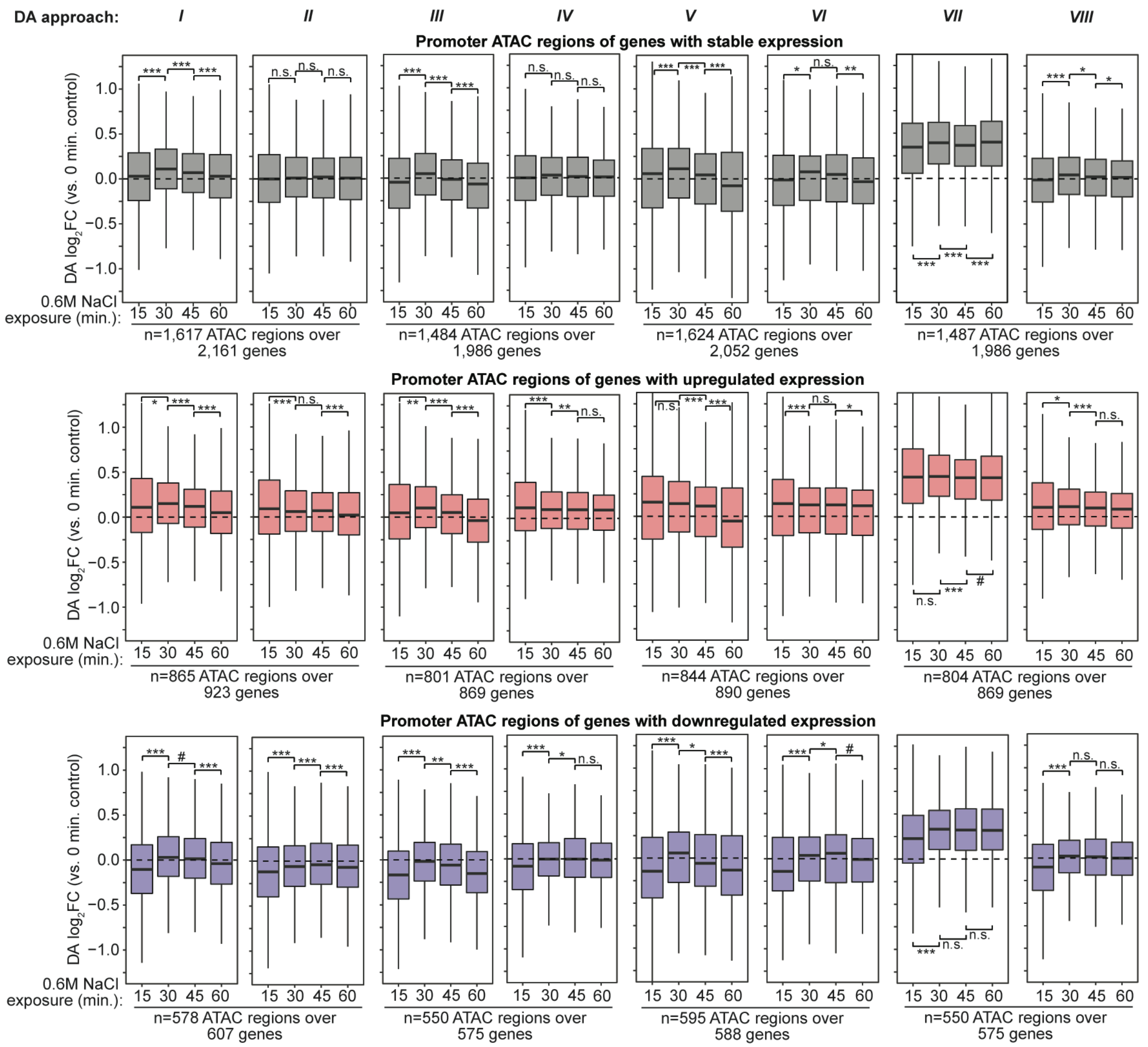

Fig. S4 | Complete statistical analysis of Schep *et al.* osmotic stress ATAC-seq time series DA methods. Boxplots in the style of Tukey without outliers for all promoter DA log_2FC measurements at each time point compared to 0 minute control, regardless of significance, separated by Ni *et al.* gene expression response classification. Top (gray) is at genes with stable expression, middle (red) is at genes with upregulated expression, and bottom (blue) is at genes with downregulated expression. Statistic is paired, two-tailed Wilcox test.

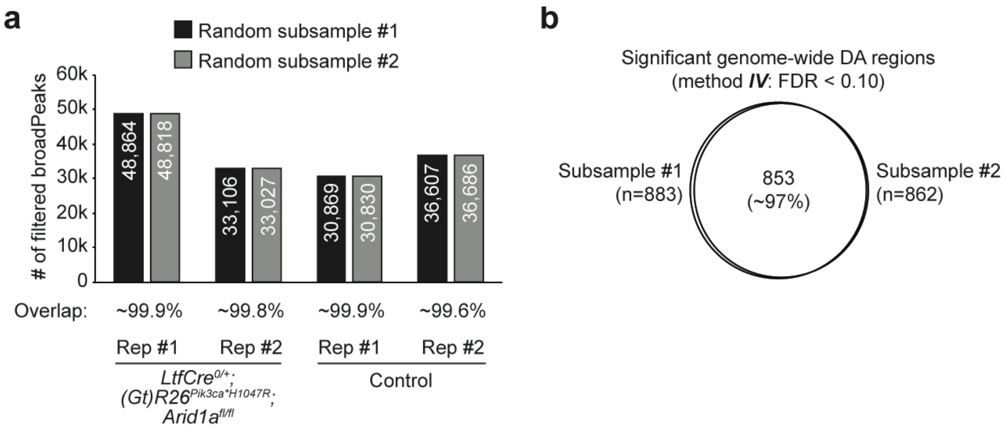

Fig. S5 | Replicated analysis downstream of random subsample seeds for complexity normalization. a, Statistics of blacklist-filtered *MACS2* broadPeak (FDR < 0.05) calls per library following two random subsamples to normalize molecular complexity. Each library retained over 99% of overlapping peaks between the two random subsample replicates. **b**, Proportional Euler diagram overlap of significant DA regions (FDR < 0.10) by method (*IV*) following the two random subsamples.

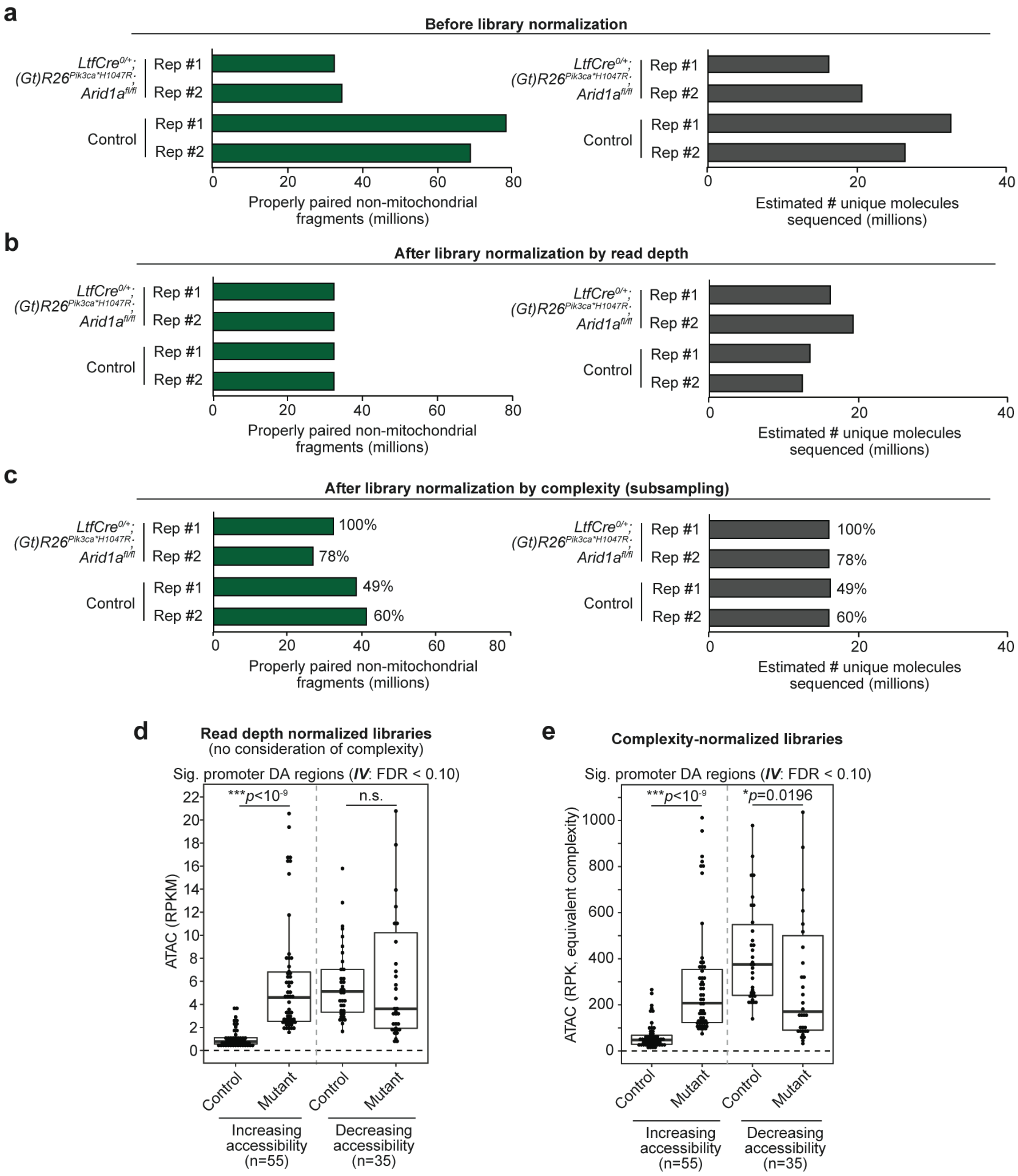

Fig. S6 | Effects of library complexity normalization by random subsampling. Library statistics from the analyzed *in vivo* mouse ATAC-seq data set by Wilson & Reske *et al.* before normalization (**a**), after read depth normalization (**b**), and after complexity normalization by subsampling (**c**). The left plots display the number of properly paired, non-mitochondrial

fragments in each library, and the right plots display the library complexities in the format of estimated number of unique molecules sequenced. Percentages listed next to each sample in **c** represent the portion of each library that was retained during subsampling. *LtfCre*$^{0/+}$*; (Gt)R26*^{Pik3ca*H1047R}*; Arid1a*$^{fl/fl}$ replicate #1 sample was estimated as the least complex library at current read depth and therefore was not subsampled. The complexity-normalized libraries were used for all subsequent analyses. **d**, quantification of ATAC signal by RPKM (i.e. read depth normalization) in control and mutant libraries, at a set of a promoter ATAC regions determined significantly DA by analysis *IV*. DA regions are further segregated by increasing and decreasing accessibility. Statistic is paired, two-tailed Wilcox test. **e**, ATAC signal at regions as in **d** but instead quantified by RPK (reads per kilobase) in libraries of equivalent molecular complexity.
